# A Community Prenatal Intervention in Social Nutrition: Evaluating the Impact on Pregnancy and Birthweight Outcomes

**DOI:** 10.3390/nu14061151

**Published:** 2022-03-09

**Authors:** Elise Carbonneau, Alex Dumas, Annie Brodeur-Doucet, Bénédicte Fontaine-Bisson

**Affiliations:** 1 School of Nutrition Sciences, University of Ottawa, Ottawa, ON K1N 6N5, Canada; bfontain@uottawa.ca; 2 Centre NUTRISS—Nutrition, Santé et Société, Institut sur la Nutrition et les Aliments Fonctionnels, Université Laval, Quebec, QC G1V 0A6, Canada; 3 School of Nutrition, Université Laval, Quebec, QC G1V 0A6, Canada; 4 School of Human Kinetics, University of Ottawa, Ottawa, ON K1N 6N5, Canada; adumas@uottawa.ca; 5 Institut du Savoir Montfort, Montfort Hospital, Ottawa, ON K1K 0T2, Canada; 6 Montreal Dietary Dispensary, Montreal, QC H3H 1J3, Canada; abdoucet@dispensaire.ca

**Keywords:** perinatal intervention, pregnancy, social nutrition, immigrant, vulnerability, gestational diabetes mellitus, gestational anemia, gestational weight gain

## Abstract

This study aims to assess the associations between structural features of the Montreal Diet Dispensary’s social nutrition intervention and pregnancy (i.e., anemia, gestational diabetes mellitus (GDM), gestational weight gain (GWG), hypertension) and birthweight outcomes (i.e., small- or large-for-gestational-age) among pregnant women, most of them recent immigrants. The study consists of a secondary analysis of the digital client database of the Montreal Diet Dispensary (*n* = 2925). Logistic regressions were used to estimate the odds of pregnancy and birthweight outcomes, depending on structural features of the intervention. Pregnant women who attended a welcoming group session presented lower odds of GDM and anemia compared to those who did not attend. A longer duration of intervention was also associated with lower odds of GDM and anemia. Each additional appointment with a dietitian was associated with higher odds of excessive GWG and lower odds of insufficient GWG only among women with a pre-pregnancy BMI lower than 25 kg/m^2^. This study emphasizes the importance of providing nutritional services early in pregnancy to reduce the risk of GDM and anemia. It also stresses the importance of using appropriate nutritional guidelines to avoid increasing the risk of excessive GWG.

## 1. Introduction

Malnutrition during pregnancy—which includes undernutrition (e.g., caloric/protein or micronutrient deficiencies) and overnutrition (e.g., excess of calories with micronutrient excess or deficiencies)—is associated with a wide range of adverse outcomes for maternal and infants’ physical, mental and neurodevelopmental health [1,2,3,4,5]. These outcomes are particularly important for young children, given that their first 1000 days of life, extending from conception to early childhood, constitute a critical period during which malnutrition and food insecurity can have serious negative consequences for their growth, development and health [6,7,8].

Improving diet quality is among the most effective and sustainable strategies to correct nutritional status and produce positive impacts on growth and development during the pre- and postnatal periods [9]. Nutrition education and counselling during pregnancy can improve nutritional status [10], decrease the risk of inadequate gestational weight gain, maternal anemia, and prematurity, and increase birthweight, to greater effect when nutrition support (e.g., food or supplements) and safety nets are also provided [11]. However, modifying dietary behaviours and intakes is difficult due to their complex, intersecting physiological, psychological, emotional, sociocultural, economic, environmental and political causes [9,12,13]. Well-known examples of perinatal nutrition programs that have been implemented to address food insecurity and malnutrition during pregnancy include the Canadian Prenatal Nutrition Program (CPNP) [14], the Special Supplemental Nutrition Program for Women, Infants, and Children (WIC) (USA) [15], and Healthy Start (UK) [16]. Studies have identified the positive impacts of perinatal nutrition programs on maternal diet and birth outcomes, including improved maternal dietary intake of healthier food (e.g., whole grains, fruits, vegetables, low-fat milk) and a small but clinically relevant increase in birthweight [17,18,19,20,21].

Despite existing programs, pregnant women of low socioeconomic status continue to display poorer nutritional status and increased adverse health outcomes compared to more affluent women [8,9,22,23,24,25]. Close attention should be paid to pregnant women who are immigrants, asylum seekers and refugees, since they have a higher risk of unfavourable perinatal health outcomes [26] and negative experiences of care and clinical intervention, including problems with communication, discrimination, poor relationships with health professionals, and cultural clashes [27]. A better understanding of how different aspects of nutritional interventions targeted to vulnerable groups impact maternal and infant outcomes may help in developing approaches that are more effective and adapted to their needs during the first 1000 days of a child’s life [9].

Founded in 1879 in Montreal (Quebec, Canada), the Montreal Diet Dispensary (MDD) is a well-established community organization advocating a social nutrition approach in providing multidimensional support to vulnerable pregnant women and their offspring [28]. In the 1960s and 1970s, Agnes C. Higgins, a dietitian and the Dispensary’s Executive Director, developed a method to prevent low birthweight in newborns by providing individualized counselling using motivational strategies, free food items to satisfy daily protein and calorie requirements (i.e., one egg, one litre of whole milk, one orange), multivitamin supplements, and regular follow-ups [29]. By proving its effectiveness, the Higgins method laid the foundation for the Canada Perinatal Nutrition Program and WIC programs. Since 2013, the MDD has delivered individual and group consultations that help between 650 and 1500 women each year, with the vast majority being immigrants with low incomes [28]. Its social nutrition approach consists of collaborative and interdisciplinary efforts adapted to the realities of low-income pregnant women, particularly recent immigrants. For example, the organization offers individual nutritional counselling adapted to the women’s cultural background, hosts various workshops (e.g., positive parenting, healthy eating), provides postnatal follow-up and breastfeeding counselling, and supplies food baskets/vouchers, prenatal multivitamin supplements and referrals to services of other community organizations [28].

Despite the MDD’s 140 years in operation and 65 years of offering services to low-income pregnant women, little information exists on the impact of the services they provide. Prior to 2000, studies documented the impact of the intervention on nutritional status and outcomes of pregnancy in, among others, adolescent mothers and the context of twin pregnancy [29,30,31,32]. More recently, Ménard et al. [33] documented the disparities in pregnancy and birth outcomes among participants in the MDD intervention, namely disparities in the prevalence of anemia, gestational diabetes mellitus (GDM), preterm birth and birth weight, according to ethnicity, immigration status, maternal age, pre-pregnancy BMI and marital status. Their results called for a deeper evaluation of the MDD intervention in order to better understand which structural features of the intervention could prevent adverse pregnancy outcomes and to prioritize higher-risk groups in this multi-ethnic, low-income population.

The objective of this study is twofold. First, it aims to assess the associations between structural features of the social nutrition intervention (i.e., attendance at a welcoming group session, number of appointments with a dietitian, duration of the intervention) and pregnancy (i.e., anemia, GDM, hypertension, gestational weight gain) and birthweight (i.e., small- or large-for-gestational-age) outcomes. Second, the study aims to identify whether these associations vary according to maternal characteristics (i.e., immigration status, age, marital status, education, pre-pregnancy BMI).

## 2. Materials and Methods

This research consists of a secondary analysis of the digital client database of the MDD from June 2013 to December 2020. Of a total of 3932 files, excluded were those who had benefited from the intervention before the establishment of the digital database (i.e., before June 2013; *n* = 469) or were enrolled for their second or third pregnancy since June 2013 (*n* = 337). For women who benefited from the MDD intervention for more than one pregnancy, only the first pregnancy was analyzed. Files were also excluded if women had registered for nutrition services after 36 weeks of gestation (*n* = 59), did not receive the standard nutrition program (i.e., did not meet with a dietitian; *n* = 90), or had multiple pregnancies (*n* = 52). Therefore, 2925 files were included in the analyses.

### 2.1. Data Collection

#### 2.1.1. Sociodemographic and Individual Characteristics

Data were collected from digital client files by a dietitian. The contents of each file included demographic and socioeconomic information on age, country of birth, number of years living in Canada, marital status, years of education, after-tax family income and household size (for comparison with annual low-income cut-offs by family size, Statistics Canada [34]), gravida, and parity. Self-reported pre-pregnancy weight and height recorded on the initial visit were used to calculate pre-pregnancy BMI.

#### 2.1.2. Nutritional Intervention

When a pregnant woman contacts the MDD, a dietitian administers a screening questionnaire composed of risk indicators (e.g., maternal age, gestational age, household income, obstetric history, health conditions, and alcohol or drug use) to assess her priority level. Based on the results of the screening questionnaire and on the dietitian’s practical judgment, the pregnant woman is assigned to a dietitian for her entire pregnancy, according to the dietitian’s expertise and availability for the first individual appointment. The interval between individual appointments varies depending on the client’s needs and the dietitian’s judgement. Beginning in September 2015, due to long waiting lists for the first appointment, welcoming group sessions were organized to meet participants before their first individual appointment with the dietitian. Welcoming group sessions were offered to all pregnant women but were not compulsory. During the session, participants were introduced to nutritional guidelines for a healthy pregnancy to increase their knowledge and perception of self-efficacy for healthy eating. Welcoming group sessions ended in 2020 when the COVID-19 pandemic limited the MDD to online meetings. The duration of the intervention corresponds to the period between the first individual appointment with the dietitian and the birth. For the analyses, the duration of intervention was separated into quartiles (Q1: ≤9.3 weeks; Q2: >9.3 to 12.1 weeks; Q3: >12.1 to 15 weeks; Q4: >15 weeks). Women in the Q4 are those who benefited from the intervention the earliest in their pregnancy. Since two clients can have the same duration of intervention but a different number of individual appointments, both independent variables were included in the statistical models. By doing so, both the duration and the intensity of the intervention received were assessed.

#### 2.1.3. Pregnancy and Birthweight Outcomes

Weight was measured at the MDD on the same scale at each follow-up visit. Total gestational weight gain (GWG) was assessed based on the last known weight before giving birth and self-reported pre-pregnancy weight and was classified as per the 2010 Institute of Medicine guidelines according to pre-pregnancy BMI [35]. GDM, anemia and hypertension were self-reported by the patient and ascertained by the dietitian, using copies of medical reports when possible. Anemia was defined as having hemoglobin of <110 g/L [36] at any time during the pregnancy. Hypertension included both pre-eclampsia and gestational hypertension, defined as having a diastolic pressure of >90 mmHg (Public Health Agency of Canada [37]).

Infants’ birthweight and gestational age at birth were retrieved from the vaccination records completed by obstetric nurses after birth. Size at birth was classified according to gestational age; the small-for-gestational-age (SGA) cut-off point was set at the 10th percentile, and the large-for-gestational-age (LGA) at the 90th percentile, according to Kramer’s growth curve reference for preterm- and term-born infants [38].

### 2.2. Statistical Analyses

Analyses were performed using the Statistical Analysis Software (SAS), version 9.4 (Copyright © 2013, SAS Institute Inc., Cary, NC, USA). Logistic regressions were used to generate odds ratios (ORs) with a 95% confidence interval (CI) for pregnancy and birthweight outcomes, depending on the structural features of the intervention. Independent variables were attendance at a welcoming group session (dichotomous variable), number of appointments with a dietitian (continuous variable), and duration of the intervention (divided into quartiles, with the first quartile being the reference). The dependent variables tested were pregnancy (i.e., GDM, anemia, hypertension, excessive and insufficient GWG) and birthweight (i.e., small- or large-for-gestational-age) outcomes. Crude models were tested (no covariate) and then adjusted for maternal age, maternal education, household income, immigration status, marital status, smoking, gravida, pre-pregnancy BMI (except for analyses related to excessive or insufficient GWG), and history of GDM or anemia (for analyses pertaining to GDM and anemia only, respectively). Interaction terms were tested to assess possible the moderation effects of sociodemographic characteristics (i.e., immigration status, age, marital status, education, pre-pregnancy BMI) on the associations between the independent (structural features of the intervention) and dependent (pregnancy and birthweight outcomes) variables. There were no missing data, except for maternal education level, which was missing for 56 participants. The mean and median values for the remaining 2869 participants were, respectively, 14.1 and 14 years of education; therefore, we used mean imputation for the missing data.

### 2.3. Process Evaluation

The MDD intervention analyzed in this study was delivered from 2013 to 2020 by approximately a dozen dietitians, who adapted their approach to the sociocultural background of each pregnant woman. Over the years, changes were made progressively to offer services tailored to the clientele. In order to properly interpret the results obtained in this study, observations were shared with three intervention providers, including senior administrators who had between 4 and 14 years of experience with the organization. Inspired by process evaluation designs [39,40,41,42], these discussions with MDD dietitians on how the intervention varies according to different contexts helped in the interpretation of the results.

## 3. Results

Characteristics of the 2925 participants included in this study are presented in Table 1. Participants were aged 15 to 52 years (mean 32.1 ± 5.3), and most of them had completed more than 11 years of formal education (78.0%), which corresponds to a high school diploma. The majority of participants were married or in a relationship (85.3%) and had an annual family income below the low-income cut-off (82.2%). Participants came mostly from Africa and Latin America. Although 68.3% of the participants had been in Canada less than 5 years, the majority of the sample either had Canadian citizenship or permanent residency (82.2%).

Participants registered at the MDD at 21.4 ± 6.9 weeks of gestation, and a total of 355 women participated in a welcoming group session at 20.9 ± 5.1 weeks (range: 7.9 to 35.6 weeks). Participants had their first individual appointment with a dietitian at 27.1 ± 4.4 weeks (range: 6.6 to 38.4 weeks) and had 4.5 ± 1.7 appointments (range: 1 to 11) during their pregnancy. The duration of intervention was 12.2 ± 4.5 weeks (range: 0.1 to 31.9 weeks).

In the study sample, the prevalence of GDM was 5.5% (*n* = 161), maternal anemia was 19.0% (*n* = 557), hypertension was 1.1% (*n* = 33), excessive GWG was 48.1% (*n* = 1408) and insufficient GWG was 22.9% (*n* = 671). The prevalence of adverse newborn birthweight outcomes was 9.3% (*n* = 272) for SGA and 9.6% (*n* = 189) for LGA.

The risk of pregnancy and birthweight outcomes according to structural features of the intervention are presented in Table 2. Attending a welcoming group session was associated with a 55% reduced risk of GDM and 34% reduced risk of anemia compared to levels for those who did not attend a welcoming group session, in adjusted models. A longer duration of intervention was also associated with lower odds of having GDM and anemia. More precisely, compared to an intervention duration in the first quartile (i.e., ≤9.3 weeks), duration in the third quartile (i.e., 12.2–15 weeks) and fourth quartile (i.e., >15 weeks) were associated respectively with a 65% and 61% reduced risk of GDM, and a 37% and 56% reduced risk of anemia, in adjusted models. However, attendance at a welcoming session and a longer duration of intervention were not associated with any other adverse pregnancy and birthweight outcomes. In the crude model, the number of appointments with a dietitian was associated with lower odds of hypertension, but the association was no longer significant when adjusted for covariates. The number of appointments with a dietitian was not significantly associated with any other pregnancy and birthweight outcomes.

Additional logistic regressions were performed to assess whether maternal characteristics (i.e., immigration status, age, marital status, education and pre-pregnancy BMI) moderated the associations between structural features of the intervention and pregnancy and birthweight outcomes. Pre-pregnancy BMI was found to significantly moderate the association between the number of appointments with a dietitian and both excessive GWG (*p* < 0.0001) and insufficient GWG (*p* < 0.0001). Further analyses were then stratified by pre-pregnancy BMI (Table 3). These analyses showed that a higher number of appointments with a dietitian was associated with higher odds of excessive GWG and lower odds of insufficient GWG among women with a pre-pregnancy BMI lower than 25 kg/m^2^, but not among women with a pre-pregnancy BMI corresponding to overweight or obesity (see Table 3). More specifically, for participants with a pre-pregnancy BMI lower than 25 kg/m^2^, each increase in the number of appointments with a dietitian is associated with an 11% increased risk of excessive GWG and an 11% reduced risk of insufficient GWG, in adjusted models. The other maternal characteristics tested were not found to significantly moderate any associations between structural features of the intervention and pregnancy and birthweight outcomes.

## 4. Discussion

The aim of this study was to better understand whether structural features of the MDD intervention influence pregnancy and birthweight outcomes and whether this is moderated by the characteristics of the low-income and/or immigrant/refugee pregnant population. Three structural features of the intervention (i.e., attendance at a welcoming group session, duration of the intervention and number of appointments with a dietitian) were analyzed in relation to GDM, anemia, hypertension, excessive and insufficient GWG, and small- or large-for-gestational-age birthweight. Although the literature on prenatal interventions is abundant, features related to the “dosage” of interventions are rarely documented, as pointed out by Beulen et al. [43] in a recent systematic review of tools to promote healthy antenatal dietary intakes.

Our results indicate that a longer duration of intervention is associated with a decreased risk of developing GDM and anemia. Participants were, on average, at 21 weeks of gestation when they registered at the MDD, and they met a dietitian for the first time, on average, at 27 weeks of gestation. This suggests that the duration of the intervention could be longer if the delay between the opening of the client file and the first appointment with a dietitian was shortened. To address this delay, which was due to an increased demand for their services, the MDD decided, in 2015, to implement a welcoming group prior to individual appointments with a dietitian. These one-time sessions introduced participants to nutritional guidelines, to increase their knowledge and perception of self-efficacy for healthy eating. Our study suggests that adding a welcoming group session to existing nutritional prenatal nutrition programs can be effective in improving maternal health outcomes.

Solutions aiming to reduce the odds of GDM and maternal anemia are compelling for the MDD, given that rates of these pregnancy issues were higher among its clientele than the national rates [33]. GDM can be responsible for a variety of perinatal complications, including congenital malformations, macrosomia, prematurity and fetal/newborn mortality [44]. Children exposed to GDM in utero are at greater risk of obesity, impaired metabolic health and neurodevelopmental delay [45,46]. Results of the present study suggest that some structural features of the MDD intervention can protect against the development of GDM. In contrast to our results, the existing literature shows limited evidence of the effectiveness of diet or exercise interventions during pregnancy in preventing GDM [47,48,49]. It is noteworthy that few of these studies include vulnerable populations (i.e., recent immigrants or low-income women). It can be hypothesized that the social nutrition approach taken by the MDD—which includes multidimensional, collaborative and interdisciplinary support adapted to the realities of low-income immigrant women—acts simultaneously on a number of vulnerability factors, resulting in empowerment, behavioural changes and positive health outcomes. More studies involving vulnerable populations are required to better understand how structural features of perinatal interventions impact the prevention of pregnancy issues such as GDM. Additionally, studies have identified stress and anxiety as potential risk factors for GDM [50,51,52]. The large majority of participants in the present study had a low household income and were therefore more likely to present high stress and anxiety levels related to economic precarity. The reduced risk of GDM we observed in association with attendance at a welcoming group session and a longer duration of intervention could thus be partly explained by potentially reduced stress and anxiety levels among participants who benefited from the earlier intervention. Since attendance at a welcoming group session was not compulsory, it can be hypothesized that participants who freely decided to attend were more motivated to improve their eating habits.

This study shows that attendance at a welcoming session as well as a longer duration of intervention are also associated with a decreased risk of anemia. Maternal anemia has been associated with higher rates of pre-eclampsia, Caesarean delivery and preterm birth, a longer hospitalization and a lower infant Apgar score [53,54]. Children whose mothers are anemic are at increased risk of developing anemia because their iron stores may be low at birth [55]. In a review that included 61 randomized or quasi-randomized trials evaluating the effects of daily oral preventive supplementation with iron, Peña-Rosas et al. [56] concluded that iron supplementation reduces the risk of maternal anemia and iron deficiency in pregnancy. Somewhat closer to the MDD intervention, Sunuwar et al. [57] observed that a 10-week intervention, including two nutrition education counselling sessions and a diet plan based on iron-rich foods during the second trimester of pregnancy, increased hemoglobin as well as knowledge about iron among mild to moderately anemic pregnant women. The reduction of anemia associated with the MDD intervention is therefore likely due to the vitamin and mineral supplements received from the first individual appointment until the birth and to the nutritional advice about iron-rich foods delivered during both the welcoming group session and the individual appointments with a dietitian.

The present study suggested that an increased number of appointments with a dietitian is associated with an increased risk of excessive GWG and a decreased risk of insufficient GWG among women who were underweight or normal weight before pregnancy. These associations were not significant among women who were overweight and obese. Historically, the MDD dietitians have had the primary mission of trying to reduce the prevalence of preterm birth and low birthweight by, among other means, encouraging pregnant women to increase their daily energy and protein intake. However, the prevalence of preterm birth and low birthweight of MDD newborns is lower than local and national rates [33], and therefore this issue should no longer be the number one clinical priority for the MDD. Discussions with MDD dietitians helped us understand that although the profile of the MDD’s clientele has changed rapidly over the last decades (higher percentage of immigrants, increased pre-pregnancy maternal overweight and obesity), the foundations of the intervention have not been evaluated since 1997, and changes in the intervention guidelines have been recent and modest. The observed association between the number of appointments with a dietitian and the higher risk of excessive GWG emphasizes the need to update the MDD intervention guidelines, particularly those related to energy and protein intake. Results of a meta-analysis of 39 cohorts including more than 260,000 births show that high GWG is associated with higher risks of gestational hypertensive disorders, GDM, LGA at birth, and preterm birth [58]. Excessive GWG has also been associated with an increased risk of childhood overweight/obesity [59]. In the present study, the increased prevalence of excessive GWG associated with the number of appointments with a dietitian did not result in a significant increase in LGA newborns, nor with hypertension and GDM. Since self-reported hypertension was documented in only 1.1% (*n* = 33) of the pregnant women, it cannot be ruled out that underreporting or a type II error may explain the lack of association. It is also possible that excessive GWG may be associated with outcomes that were not available in the digital files or have a longer-term effect on mothers and children. In the post-partum period, body dissatisfaction and unrealistic weight loss goals are very prevalent and are worst among women who gained more weight during pregnancy [60]. Findings by Lovering et al. [61] suggest that, during the post-partum period, women experience strong sociocultural pressure to attain unrealistic body shapes/sizes, which contributes to body image concerns. Associations between poor body image and a higher prevalence of post-partum depression have also been documented in recent studies [62,63]. Thus, the higher risk of excessive GWG associated with the number of appointments with a MDD dietitian should not be taken lightly, given the potential short- and long-term negative effects it may have on maternal and offspring health. Since 2017, changes have been gradually applied to the method, particularly with regard to the calculation of energy needs, which demonstrates that this is already a concern for the MDD organization. This calls for a replication of the present study to evaluate the potential impacts of these changes on pregnancy outcomes. Future studies are also needed to assess the level of knowledge that pregnant women gain during the intervention, notably regarding GWG. Such a study would allow the evaluation of the MDD’s objective of increasing women’s empowerment and parenting self-efficacy.

Only a few studies have assessed the impacts of the duration of a prenatal nutrition intervention on pregnancy and birth outcomes. Stockbauer [64] suggested that the WIC program delivered in Missouri in 1982 had to last at least 7 months before a gain in birthweight (+50g) and a reduction of 18% in low birthweight prevalence were observed. In the present study, the duration of intervention was not associated with SGA. However, in the present study’s sample, only 1% of the participants benefited from an intervention that lasted more than 6 months. Some associations between features of the intervention and birthweight might have been observed if the intervention had lasted longer. However, it is noteworthy that Ménard, Sotunde and Weiler [33] observed a lower prevalence of SGA at the MDD compared to local and national rates. Therefore, there may not be much room for improvement.

Major strengths of the present study lie in the uniqueness of the evaluation of a real-life perinatal intervention that took place over a 7-year period and involved nearly 3000 vulnerable pregnant women. The use of the anonymized database for the purpose of secondary analysis limited potential bias related to recruitment and allowed an evaluation of the intervention as it is normally provided to pregnant women. These strengths are not, however, without limitations. In order to respond to their clients’ needs, dietitians have to personalize the intervention accordingly. Therefore, the intervention is not standardized, making it more difficult to target its features at a granular level in relation to the outcomes under study. In addition, most information retrieved from the clients’ files was self-reported, and details leading to the medical diagnoses were not always available (e.g., results of oral glucose tolerance tests). Another limitation of the study is the absence of a comparison group, preventing inference of the relative effectiveness of the intervention compared to another intervention or to no intervention. Finally, because of the unique history and context of the intervention offered by the MDD, researchers should be cautious about applying these results to other low-income populations or perinatal nutrition interventions.

## 5. Conclusion

In conclusion, this assessment of a community prenatal intervention in social nutrition highlights the importance of providing nutritional services early in pregnancy to reduce the risk of GDM and anemia. This study also stresses the importance for perinatal nutrition organizations to encourage their dietitians to use their clinical judgement and not rely solely on energy needs during nutrition counseling, in order to avoid increasing the risk of excessive weight gain during pregnancy. This study emphasizes the usefulness of detailed client databases for the purposes of evaluation, which can help organizations to develop or improve their nutritional interventions.

## Figures and Tables

**Table 1:** Characteristics of the study population (*n* = 2925).

Characteristics	*n*	%
Maternal age (years)		
<20	42	1.4
20–34	2017	69.0
35–39	673	23.0
≥40	193	6.6
Education (years)		
<11	283	9.7
11	304	10.4
12–13	573	19.6
≥14	1709	58.4
Missing data	56	1.9
Marital status		
Single/divorced	429	14.7
With a partner	2496	85.3
Income		
Below low-income measure *	2404	82.2
Maternal region of origin		
Middle East and North Africa	935	32.0
Sub-Saharan Africa	638	21.8
Latin America and Caribbean	612	20.9
East Asia and Pacific	296	10.1
North America **	287	9.8
Europe and Central Asia	80	2.7
South Asia	77	2.6
Immigration status		
Canadian citizen or permanent resident	2404	82.2
Visa/refugee/awaiting status	521	17.8
Period living in Canada		
Canadian born	282	9.6
Established immigrants (>10 years)	254	8.9
Recent immigrant (5–10 years)	390	13.3
Very recent immigrant (<5 years)	1999	68.3
Pre-gravida body mass index		
Underweight (<18.5 kg/m^2^)	124	4.2
Normal (18.5–24.9 kg/m^2^)	1409	48.2
Overweight (25.0–29.9 kg/m^2^)	898	30.7
Obese (≤30.0 kg/m^2^)	494	16.9

Notes. * Low-income cut-offs after tax by family size; Statistics Canada (2021). ** Includes Canada and United States.

**Table 2:** Odds ratio of adverse pregnancy and birthweight outcomes according to structural features of the intervention.

	GDM *		Anemia **		Hypertension ^†^		Excessive GWG ^±^		Insufficient GWG ^±^		SGA ^†^		LGA ^†^	
	OR	95% CI	OR	95% CI	OR	95% CI	OR	95% CI	OR	95% CI	OR	95% CI	OR	95% CI
Welcoming group session														
Cr	0.54	0.28–1.04	0.69	0.50–0.95	0.52	0.12–2.19	1.08	0.86–1.35	1.00	0.76–1.31	1.02	0.70–1.51	1.03	0.80–1.50
Adj	**0.45**	**0.22–0.89**	**0.66**	**0.47–0.93**	0.44	0.10–1.87	1.09	0.87–1.37	1.02	0.77–1.33	1.13	0.77–1.68	0.94	0.64–1.38
Duration of the intervention														
Q1	1.00	Ref	1.00	Ref	1.00	Ref	1.00	Ref	1.00	Ref	1.00	Ref	1.00	Ref
Q2														
Cr	0.70	0.46–1.08	0.94	0.73–1.22	2.10	0.83–5.30	1.01	0.81–1.26	0.83	0.64–1.07	0.88	0.51–1.27	1.22	0.85–1.77
Adj	0.66	0.41–1.04	0.94	0.72–1.24	2.07	0.82–5.24	1.01	0.81–1.25	0.82	0.63–1.07	0.86	0.59–1.25	1.26	0.87–1.84
Q3														
Cr	0.38	0.22–0.64	0.63	0.47–0.84	1.42	0.47–4.34	1.22	0.89–1.42	0.85	0.64–1.12	0.66	0.44–1.00	1.08	0.72–1.61
Adj	**0.35**	**0.20–0.63**	**0.63**	**0.46–0.86**	1.39	0.46–4.26	1.11	0.88–1.40	0.86	0.65–1.14	0.67	0.44–1.01	1.05	0.70–1.58
Q4														
Cr	0.44	0.24–0.80	0.44	0.31–0.63	1.43	0.39–5.22	0.97	0.74–1.27	1.25	0.91–1.71	0.84	0.53–1.34	1.27	0.80–2.00
Adj	**0.39**	**0.20–0.76**	**0.44**	**0.30–0.64**	1.45	0.39–5.34	0.91	0.69–1.20	1.23	0.90–1.70	0.79	0.49–1.27	1.29	0.81–2.05
Appointments with the dietitian														
Cr	1.01	0.89–1.15	1.00	0.94–1.08	0.74	0.57–0.97	1.04	0.99–1.10	0.94	0.88–1.00	1.06	0.96–1.34	0.96	0.87–1.05
Adj	1.00	0.87–1.15	1.01	0.93–1.09	0.76	0.59–1.00	1.04	0.99–1.10	0.93	0.87–1.00	1.05	0.95–1.16	0.97	0.88–1.07

Notes: Bold font indicates statistical significance. Adj, adjusted; CI, confidence interval; Cr, crude; GDM, gestational diabetes mellitus; GWG, gestational weight gain; LGA, large for gestational age; OR, odds ratio; SGA, small for gestational age. * ORs adjusted for maternal age, maternal education level, household income, immigration status, marital status, smoking, gravida, pre-gravida body mass index and history of GDM. ** ORs adjusted for maternal age, maternal education level, household income, immigration status, marital status, smoking, gravida, pre-gravida body mass index and history of anemia. ^±^ ORs adjusted for maternal age, maternal education level, household income, immigration status, marital status, smoking, gravida and pre-gravida body mass index. ^†^ ORs adjusted for maternal age, maternal education level, household income, immigration status, marital status, smoking and gravida.

**Table 3:** Odds ratio of inadequate gestational weight gain (GWG) according to structural features of the intervention, stratified according to pre-pregnancy BMI categories.

	BMI < 25 kg/m^2^				BMI ≥ 25 kg/m^2^			
	Excessive GWG		Insufficient GWG		Excessive GWG		Insufficient GWG	
	OR	95% CI	OR	95% CI	OR	95% CI	OR	95% CI
Welcoming group session								
Cr	1.16	0.83–1.60	0.90	0.62–1.32	0.92	0.67–1.26	1.19	0.81–1.75
Adj	1.19	0.85–1.66	0.90	0.61–1.32	0.92	0.67–1.27	1.20	0.81–1.77
Duration of the intervention								
Q1	1.00	Ref	1.00	Ref	1.00	Ref	1.00	Ref
Q2								
Cr	1.13	0.82–1.55	0.73	0.52–1.02	0.86	0.62–1.19	1.03	0.68–1.57
Adj	1.12	0.82–1.54	0.72	0.51–1.01	0.86	0.62–1.19	1.00	0.66–1.53
Q3								
Cr	1.20	0.86–1.68	0.78	0.54–1.12	0.92	0.66–1.30	1.05	0.68–1.63
Adj	1.18	0.84–1.66	0.78	0.54–1.12	0.91	0.64–1.28	1.04	0.67–1.61
Q4								
Cr	1.00	0.68–1.48	1.28	0.85–1.92	0.80	0.54–1.20	1.34	0.81–2.22
Adj	0.89	0.60–1.33	1.27	0.84–1.92	0.80	0.53–1.21	1.24	0.75–2.07
Appointments with the dietitian								
Cr	1.10	1.01–1.19	0.90	0.82–0.98	1.05	0.96–1.14	0.97	0.87–1.08
Adj	**1.11**	**1.02–1.20**	**0.89**	**0.84–0.97**	1.05	0.96–1.14	0.98	0.88–1.09

Notes: Bold font indicates statistical significance. Adj, adjusted; CI, confidence interval; Cr, crude; GWG, gestational weight gain; OR, odds ratio. ORs adjusted for maternal age, maternal education level, household income, immigration status, marital status, smoking and gravida.

## Data Availability

The data presented in this study are available on reasonable request from the corresponding author.
